# Effects of neighboring transitions on the mechanisms of electromagnetically induced absorption and transparency in an open degenerate multilevel system

**DOI:** 10.1038/s41598-021-04038-5

**Published:** 2022-01-07

**Authors:** Zeeshan Ali Safdar Jadoon, Heung-Ryoul Noh, Jin-Tae Kim

**Affiliations:** 1 Department of Photonic Engineering, Chosun University, Gwangju, 61452 Korea; 2 Department of Physics, Chonnam National University, Gwangju, 61186 Korea

**Keywords:** Atomic and molecular physics, Optical physics

## Abstract

In this study, optical Bloch equations with and without neighboring hyperfine states near the degenerate two-level system (DTLS) in the challenging case of $^{85}$Rb D2 transition, which involves the Doppler broadening effect, are solved. The calculated spectra agree well with the experimental results obtained based on the coupling-probe scheme with orthogonal linear polarizations of the coupling and probe fields. The mechanisms of electromagnetically induced absorption (electromagnetically induced transparency) for the open $F_g=3 \rightarrow F_e=2$ and 3 transitions (open $F_g=2 \rightarrow F_e=2$ and 3 transitions) are determined to be the effect of the strong closed $F_g=3 \rightarrow F_e=4$ transition line (strong closed $F_g=2 \rightarrow F_e=1$ transition line); this finding is based on a comparison between the calculated absorption profiles of the DTLS without neighboring states and those of all levels with neighboring states, depending on the coupling and probe power ratios. Furthermore, based on the aforementioned comparison, the crucial factors that enhance or reduce the coherence effects and lead to the transformation between electromagnetically induced absorption and electromagnetically induced transparency, are (1) the power ratios between the coupling and probe beams, (2) the openness of the excited state, and (3) effects of the neighboring states due to Doppler broadening in a real atomic system.

## Introduction

Since the initial reports on electromagnetically induced absorption (EIA)^1^ and electromagnetically induced transparency (EIT)^2,3^, numerous studies^4–19^ have been conducted on the transition between EIT and EIA and the mechanisms of these transitions, which result from quantum coherence and interference between coupled atomic states in coupling-probe and Hanle-type experiments. However, the mechanism of transition between EIAs and EITs in terms of powers and the polarization configurations of the coupling and probe beams, the openness of the transitions, and neighboring effects with Doppler broadening remain unclear^4–19^. The mechanisms of EIA transition from EIT depending on the coupling and probe powers in the case of the $F_e=F_g-1$ open system of the $^{85}$Rb D2 line have not been sufficiently elucidated, and such EIAs at weak coupling and probe powers have not been observed to date.

Lezama^20^ established three necessary EIA conditions for a degenerate two-level system (DTLS) based on initial investigations of EIA resonance^1^ using coupling and probe lasers: (1) the ground state must be degenerate; (2) $F_e=F_g+1$; (3) the $F_g \rightarrow F_e$ transition must be closed, without considering the contributions to absorption or transmission coherence strength from neighboring states embedded in the Doppler broadening profiles, the power ratios between the coupling and probe beams, the polarization configurations, and the openness of the excited state.

In-depth studies have been conducted on EIAs in a DTLS that partially violate the three aforementioned conditions^4–6,8–17,19^; similarly, studies have also focussed on EITs in a DTLS that partially satisfy these conditions^7,9,10,18^ have been performed. Thus, the aforementioned conditions for EIAs are satisfied in specific scenarios such that EIA and EIT can be observed depending on the coupling and probe powers, the openness of the transition, the polarization configurations, and effects of neighboring states in the cases of $F_e=F_g+1$, $F_e=F_g$, and $F_g-1$.

Several studies have also been conducted on the transitions between EIA and EIT depending on the general EIA conditions of the $F_e=F_g+1$ open system^4–7,12,14–17^. However, expected EITs in the DTLS in the weak power regime and in the case of $F_e \le F_g$, which violate the established EIA conditions^20^, transforming into genuine and symmetric EIAs considering the Doppler broadening effect have still not been reported. The first EIA resonances in the case of $F_e \le F_g$ of the D1 and D2 transitions of $^{85,87}$Rb were observed in the Hanle configuration, without any theoretical calculations for bright fluorescence profiles; these resonances violate condition (3) associated with closed transitions^4^.

Kim et al.^8^ subsequently reported EIA resonances without a theoretical analysis in open systems of the D1 lines of $^{85}$Rb and $^{87}$Rb, violating conditions (2) and (3). These EIA results were obtained only from strong coupling and probe powers exceeding the saturation power observed by several other groups^5,8,12,13,15–17^; they result from enhanced absorption governed by strong coupling and probe powers and are different from the EIAs for the same transition configuration of $F_e=F_g-1$ and $F_g$ observed in this study using weak coupling and probe powers.

Chou et al.^11^ qualitatively interpreted these EIA anomalies in open systems using a crude model in a high-power regime without a clear quantitative explanation. Zibrov et al.^12^ also observed EIA (EIT) resonances at open transitions $F_e=F_g+1$ of the D1 and D2 lines of an $^{87}$Rb atom upon artificially shifting the laser frequency toward the blue (red) end from the center of the Doppler profile in both Hanle and coupling-probe experiments, except in the cases of $F_e=F_g-1$ and $F_e=F_g$. Zhao et al.^13^ observed EIA resonances in the strong coupling-probe regime for closed and open D2 transitions $F_g=4 \rightarrow F_e=3,4$, and 5 of Cs in a coupling-probe experiment with an additional laser; they referred to Goren et al.’s theoretical analysis^14^ for the EIA anomalies at open transitions without considering the effects of neighboring states.

Auzinsh et al. observed EIA resonances in an atomic vapor cell in the case of the open $F_e=F_g+1$ transition of $^{85,87}$Rb-D1 lines^16^. Strong coupling and probe frequencies with powers greater than 100 mW/cm$^2$ were blue-shifted to escape the neighboring dark resonance in the Hanle experiment, wherein only strong coupling and probe powers govern the absorption phenomenon. The researchers also confirmed that the resonance fluorescence observed in the extremely thin cell that escaped neighboring effects owing to the Doppler broadening could not reveal such EIA resonances^17^. Auzinsh et al. also observed sign reversals of bright resonances into dark resonances at closed transitions of the D2 lines of $^{85}$Rb and $^{87}$Rb owing to high power and temperature in the Hanle experiment^18^.

Grewal et al.^19^ reported on the influence of closed neighboring hyperfine levels with strong EIA features owing to the increased ellipticities of the polarizations of coupling and probe lasers at the $F_e \le F_g$ open transitions of the D2 lines of $^{87}$Rb in the case of the Hanle configuration. However, they could not theoretically explain the absorption profiles, because of the computational complexity involved when considering the Doppler effect. Calculating the absorption profiles for D2 transitions of $^{85}$Rb has been proven to be particularly challenging because of the strong neighboring effects of Doppler profiles; in such cases, the total absorption of D2 transition lines should be calculated while considering the Doppler effect and all hyperfine levels of the ground and excited states.

In this study, we deduce that clear explanations for the transition of EIT into EIA in the case of $^{85}$Rb D2 transition can be obtained based on a comparison of the calculated absorption profiles of the DTLS without neighboring states and all energy levels of neighboring states, with respect to the power ratios between the coupling and probe powers. To investigate the effects of power on coherence, a coupling-probe experiment with two orthogonal linear polarizations from a single laser combined with two acousto-optic modulators (AOMs) is more suitable than a Hanle-type experiment, because the power ratios of the coupling and probe lasers or a wide scan of the frequency cannot be obtained in the latter.

## Theoretical calculation

**Figure 1:**
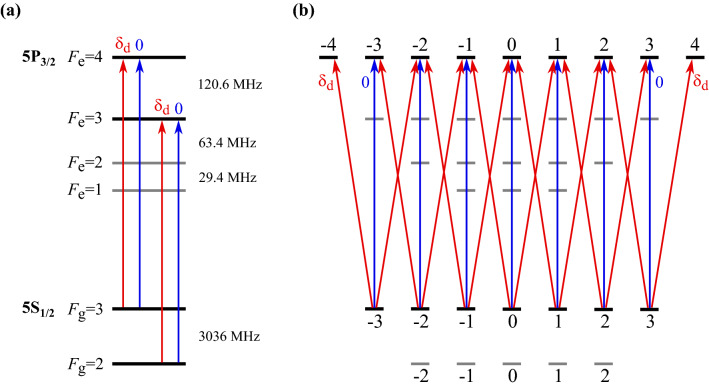
(**a**) Energy-level diagram of D2 transition lines of an $^{85}$Rb atom, wherein the red and blue lines indicate transitions by the probe and coupling beams, respectively. (**b**) Transition scheme with two linearly polarized coupling and probe beams in the case of $F_g=3 \rightarrow F_e= 4$ transition, where “0” (blue lines) and “$\delta _d$” (red lines) denote the transitions excited by the coupling and probe beams, respectively. To avoid confusion, the excitations for the $F_g=3 \rightarrow F_e = 2$ and 3 transitions are not shown.

In this section, we present a method for calculating EIA spectra while considering the neighboring effect. As the details of the calculation have been reported previously^21–25^, we describe the calculation method briefly. The energy-level diagram of the D2 transition line of $^{85}$Rb atoms is presented in Fig. 1. The coupling and probe beams that are linearly polarized with orthogonal directions propagate in the same directions. The Rabi frequency and effective detuning of the coupling (probe) beam are denoted by $\Omega _c$ ($\Omega _p$) and $\delta _c$ ($\delta _p$), respectively. The effective detunings are expressed as $\delta _c = d_c -k v$ and $\delta _p = d_p -k v$, where $d_c$ ($d_p$) denotes the frequency detuning of the coupling (probe) beam, $k (=2 \pi /\lambda )$ is the wave vector, $\lambda$ is the resonance wavelength of the lasers, and *v* is the atomic velocity. Here, $d_c$ and $d_p$ are the detunings relative to the resonance transition line under consideration.

Because the energy separation between the hyperfine states in the $5S_{1/2}$ ground state is approximately six times the Doppler linewidth of the atoms, we can calculate the EIA spectra for the $F_g =3 \rightarrow F_e =2,3$, and 4 transitions and for the $F_g =2 \rightarrow F_e =1,2$, and 3 transitions separately. In the description of the calculation method, the $F_g =3 \rightarrow F_e =2,3$, and 4 transitions are assumed. In a frame rotating with the coupling beam’s frequency, the density matrix describing the internal dynamics of the atoms is

1 $$\begin{aligned} {\dot{\rho }} = -\frac{i}{\hbar } \left[ H_0 +V,\rho \right] +{{\dot{\rho }}}_{\mathrm{relax}}, \end{aligned}$$

where $\rho$ is the density operator, and $H_0$ (*V*) is the atomic (interaction) Hamiltonian.

In Eq. (1), the atomic Hamiltonian is

2 $$\begin{aligned} H_0= & {} -\sum _{m=-4}^4 \hbar \delta _c \vert F_e =4, m \rangle \langle F_e =4, m \vert \nonumber \\&-\sum _{m=-3}^3 \hbar \left( \delta _c +\Delta _{43} \right) \vert F_e =3, m \rangle \langle F_e =3, m \vert \nonumber \\&-\sum _{m=-2}^2 \hbar \left( \delta _c +\Delta _{42} \right) \vert F_e =2, m \langle F_e =2, m \vert , \end{aligned}$$

where $\Delta _{4j}$ is the frequency spacing between the hyperfine states $\vert F_e =4 \rangle$ and $\vert F_e =j \rangle$ ($j=2$ and 3). In Eq. (1), the interaction Hamiltonian is expressed as

3 $$\begin{aligned} V= & {} \frac{\hbar }{2} \Omega _p e^{-i \delta _d t} \sum _{q=-1}^{1} \sum _{F_e =2}^4 \sum _{m=-3}^3 a_q C_{3,m}^{F_e , m+q} \vert F_e ,m+q \rangle \langle F_g , m \vert \nonumber \\&+ \frac{\hbar }{2} \Omega _c \sum _{F_e =2}^4 \sum _{m=-3}^3 C_{3,m}^{F_e , m} \vert F_e ,m \rangle \langle F_g , m \vert +\mathrm{h.c.}, \end{aligned}$$

where $F_g =3$, and h.c. denotes the Hermitian conjugate. Because the direction of the electric field of the coupling beam is selected as the quantization axis, $a_{\pm 1} =\mp 1/\sqrt{2}$ and $a_0 =0$ in Eq. (3), where $C_{F_g, m_g}^{F_e, m_e}$ is the normalized transition strength between states $\vert F_e,m_e \rangle$ and $\vert F_g,m_g \rangle$^22^, and $\delta _d ( \equiv \delta _p - \delta _c = d_p -d_c )$ is the difference between the detunings of the probe and coupling beams. In Eq. (1), ${{\dot{\rho }}}_{\mathrm{relax}}$ represents the terms related to the relaxation mechanism, such as the spontaneous emission and transit time decay^23,24^. Notably, we used a transit-time relaxation method while considering the finite interaction time between the atoms and laser light in the calculation^24^.

As reported previously^21–25^, the density matrix elements are decomposed into various oscillation components. In the case of the orthogonal linear polarization configuration, the coupling and probe beams excite the transitions with $\Delta m =0$ and $\Delta m= \pm 1$, respectively, where $\Delta m$ is the difference in the magnetic quantum number between the sublevels under consideration. In Fig. 1, “0” and “$\delta _d$” indicate the transitions excited by the coupling and probe beams, respectively. As stated earlier, $\delta _d$ is the effective frequency of the probe beam relative to the frequency of the coupling beam. The method of determining the oscillation frequencies of the density matrix elements for the orthogonal linear polarization configuration have been described in detail elsewhere^25^. Herein, only an explicit expansion of the density matrix elements responsible for the probe absorption is provided:

4 $$\begin{aligned}&\rho _{e_{m \pm 1}^{F_e},g_{m}}=\rho _{e_{m\pm 1}^{F_e},g_m}^{(1)} e^{-i \delta _d t} +\rho _{e_{m\pm 1}^{F_e},g_m}^{(2)}e^{i \delta _d t} , \end{aligned}$$

where the simplified notation for the density matrix elements is

$$\begin{aligned} \rho _{e_{m_e}^{F_e},g_{m_g}} \equiv \langle F_e ,m_e \vert \rho \vert F_g =3, m_g \rangle . \end{aligned}$$

We considered all the density matrix elements between the sublevels that are connected via a maximum of three photons. For example, the connection between the states $\vert F_e=4, m_e=m \pm 4 \rangle$ and $\vert F_g=4, m_g=m \rangle$ is neglected because at least five photons are needed to connect these two states. In addition, only two oscillation frequencies ($\pm \delta _d$) exist in the density matrix elements in Eq. (4) that connect the states $\vert F_e, m_e=m \pm 1 \rangle$ and $\vert F_g, m_g=m \rangle$. When we consider the five-photon interactions, the density matrix elements in Eq. (4) would have one more oscillation frequency ($-3\delta _d$). The absorption coefficient of the probe beam is thus expressed as

5 $$\begin{aligned} \alpha= & {} -\frac{3\lambda ^2}{2 \pi } \frac{N_{\mathrm{at}}}{\Omega _p} \int _{-\infty }^{\infty } \frac{\mathrm{d}v}{\sqrt{\pi }v_{\mathrm{mp}}} e^{-\left( v/v_{\mathrm{mp}} \right) ^2} \nonumber \\&\times \mathrm{Im} \left[ \sum _{F_e =2}^4 \sum _{q=-1}^1 \sum _{m=-3}^{3} a_q^{*} C_{3,m}^{F_e, m+q} \rho _{e_{m+q}^{F_e},g_{m}}^{(1)} \right] , \end{aligned}$$

where $N_{\mathrm{at}}$ is the atomic number density in the cell, and $v_{\mathrm{mp}}$ is the most probable speed in the cell. The parameters used in the calculation are as follows: the power of the probe beam is 15 $\upmu$W and that of the coupling beam is 50 $\upmu$W or 4 mW; the diameter of the laser beams is 4 mm, and the temperature of the vapor cell is 20 $^{\circ }$C.

## Results

**Figure 2:**
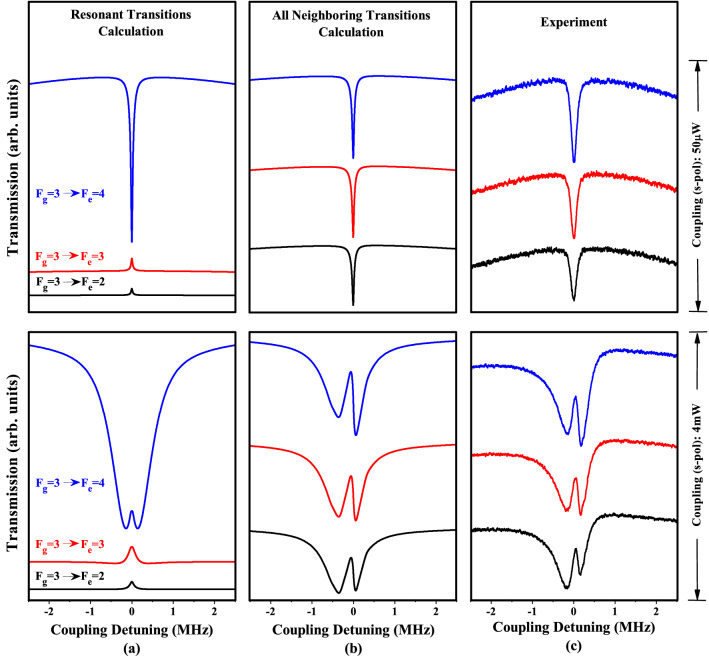
Comparison of calculated and measured spectra considering (**a**) pure two-level resonant transitions, i.e., $3 \rightarrow 2'$, $3 \rightarrow 3'$ and $3 \rightarrow 4'$; (**b**) a transition resonant at $3 \rightarrow 2'$ with neighboring hyperfine transitions of $3 \rightarrow 3'$ and $4'$, a transition resonant at $3 \rightarrow 3'$ with neighboring transitions of $3 \rightarrow 2'$ and $4'$, and a transition resonant at $3 \rightarrow 4'$ with neighboring transitions of $3 \rightarrow 2'$ and $3'$; (**c**) experimentally measured spectra for resonant transitions $3 \rightarrow 2'$, $3 \rightarrow 3'$, and $3 \rightarrow 4'$.

**Figure 3:**
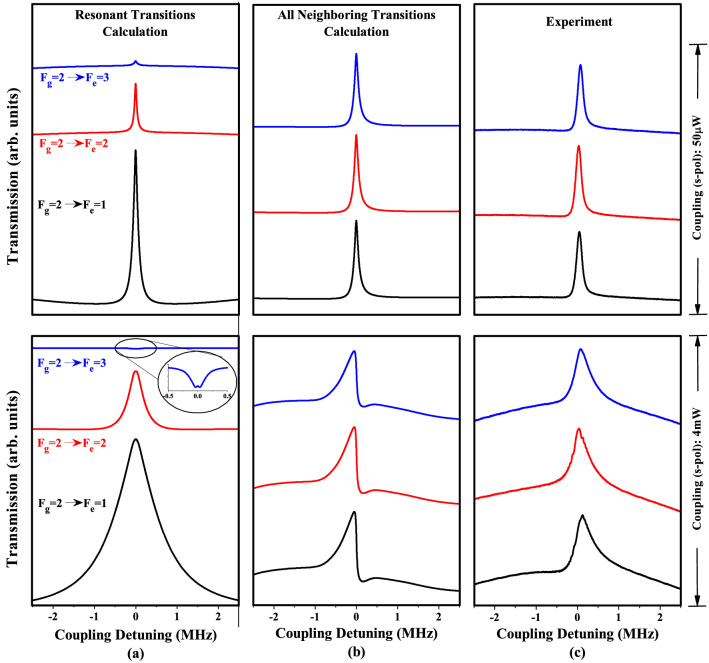
Comparison of calculated and measured spectra considering (**a**) pure two-level resonant transitions, i.e., $2 \rightarrow 1'$ , $2 \rightarrow 2'$, and $2 \rightarrow 3'$; (**b**) transition resonant at $2 \rightarrow 1'$ with neighboring hyperfine transitions of $2 \rightarrow 2'$ and $3'$, a transition resonant at $2 \rightarrow 2'$ with neighboring transitions of $2 \rightarrow 1'$ and $3'$, and a transition resonant at $2 \rightarrow 3'$ with neighboring transitions of $2 \rightarrow 1'$ and $3'$; (**c**) experimentally measured spectra for resonant transitions $2 \rightarrow 1'$, $2 \rightarrow 2'$, and $2 \rightarrow 3'$.

We compare the experimental results with the theoretical results obtained considering the resonant and all neighboring transitions resulting in EIAs and EITs. We theoretically and experimentally investigate the resonance spectra for two spectrally unresolved groups of hyperfine transitions of the $^{85}$Rb D2 lines, i.e., $F_g=3 \rightarrow F_e=2, 3$, and 4 and $F_g=2 \rightarrow F_e=1, 2$, and 3, as shown in Figs. 2 and 3, respectively. The probe and coupling beams are linearly polarized in orthogonal directions. The power of the probe beam is 15 $\upmu$W and that of the coupling beam is 50 $\upmu$W or 4 mW. The probe laser frequency is locked to a desired transition line, and the coupling laser frequency is scanned around each transition line. For simplicity, we use simplified notations for the transitions; e.g., the $F_g= 3 \rightarrow F_e =4$ transition is expressed as $3 \rightarrow 4'$, where the unprimed (primed) integers represent the angular momentum quantum numbers in the ground (excited) states.

Figure 2 presents a comparison of the calculated spectra considering (a) a pure DTLS with transitions resonant at $3 \rightarrow 2'$, $3 \rightarrow 3'$, and $3 \rightarrow 4'$; (b) a transition resonant at $3 \rightarrow 2'$ with neighboring hyperfine transitions of $3 \rightarrow 3'$ and $4'$, a transition resonant at $3 \rightarrow 3'$ with neighboring hyperfine transitions of $3 \rightarrow 2'$ and $4'$, and a transition resonant at $3 \rightarrow 4'$ with neighboring hyperfine transitions of $3 \rightarrow 2'$ and $3'$; (c) experimentally measured spectra with transitions resonant at $3 \rightarrow 2'$, $3 \rightarrow 3'$, and $3 \rightarrow 4'$ for two different weak (50 $\upmu$W) and strong (4 mW) coupling powers, as shown in the top and bottom traces, respectively.

First, we explain the cases of a weak coupling power, as shown in the top trace of Fig. 2. In the pure two-level calculations on the left side of the top trace, a closed $3 \rightarrow 4'$ transition satisfies all three necessary EIA conditions^20^ and presents a relatively strong EIA amplitude with a linewidth of 57 kHz, as expected. For open transitions $3 \rightarrow 3'$ and $3 \rightarrow 2'$, EIT spectra with small amplitudes and a linewidth resolution of 46 kHz and 41 kHz, respectively, are obtained. Considering all the neighboring states near the resonant transitions of $3 \rightarrow 2'$, $3 \rightarrow 3'$, and $3 \rightarrow 4'$, as shown in the upper trace of Fig. 2b, all the resonant transitions exhibit strong EIAs with similar amplitudes owing to a nearby closed $3 \rightarrow 4'$ transition. The calculated spectra in Fig. 2b exhibit trends strikingly similar to those observed for the measured spectra in Fig. 2c, although the observed spectra are wider than the calculated ones. Measured ultra-narrow EIA dips at a low coupling power are resolved with linewidths of 163 kHz, 156 kHz, and 167 kHz; in contrast, the linewidths obtained using all the neighboring transitions calculation are 60 kHz, 60 kHz, and 58 kHz at the $3 \rightarrow 4',3'$, and $2'$ transitions, respectively.

Second, we explain the case of a strong coupling power, as shown in the bottom trace of Fig. 2. In pure two-level calculations on the left side of the bottom trace, a closed $3 \rightarrow 4'$ transition satisfies all three necessary EIA conditions^20^ and indicates a relatively strong EIA amplitude with a wide linewidth of 1.06 MHz; an EIA dip splits into two broad dips at the center of the spectra, resulting in the emergence of a narrow EIT-like peak with a 117 kHz linewidth resolution. These EIA spectra at a strong coupling power is attributed to the contributions of Autler-Townes effects in case of orthogonal polarizations; this results in two identical EIA spectra separated at the right center after thermal averaging^21^. It should be noted that Autler-Townes splitting also exists in the absence of Doppler broadening^26^. EIT spectra with small amplitudes and wide linewidths of 194 kHz and 122 kHz for open transitions $3 \rightarrow 3'$ and $3 \rightarrow 2'$, respectively, are obtained, as shown in the lower traces in Fig. 2a.

Including all the other adjacent transitions, such a symmetric EIA is transformed into asymmetric line profiles owing to the strong neighboring effects, which differ in the red and blue detuning regions. Considering all the neighboring states near the resonant transitions of $3 \rightarrow 2'$, $3 \rightarrow 3'$, and $3 \rightarrow 4'$, as shown in the lower trace in Fig. 2b, owing to a nearby closed $3 \rightarrow 4'$ transition, all the resonant transitions exhibit strong EIAs with similar amplitudes, and remarkably similar trends in the spectra compared with the measured spectra for the closed $3 \rightarrow 4'$ transition. Asymmetric dips due to two separated EIAs at a strong power in the calculation (the measurement) are broad—with linewidths of 870 kHz, 848 kHz, and 842 kHz (607 kHz, 606 kHz, and 603 kHz)—in the red detuning and are narrow with linewidths of 297 kHz, 292 kHz, and 287 kHz (439 kHz, 402 kHz, and 377 kHz)—in the blue detuning at the $3 \rightarrow 4'$, $3'$, and $2'$ transitions, respectively. A down-shift in relative strength of the amplitude is evident from the narrow blue detuned side at the $3 \rightarrow 4'$ transition to the broad red detuned side at the $3 \rightarrow 2'$ transition because of the frequency shifts toward those transitions.

The amplitude of the total spectra across the $3 \rightarrow 4'$, $3'$, and $2'$ transitions decreases with $F_e$. The theoretical model that considers all neighboring hyperfine states (the lower trace in Fig. 2b) and the experimental results (the lower trace in Fig. 2c) agree well in terms of contrast and asymmetry. However, there are deviations in the linewidths of approximately 100 kHz in both power regimes; these can be attributed to the effect of the magnetic field, imperfect alignment of laser beams, and other effects that are not included in the calculation.

We further analyze the spectrally unresolved group of hyperfine transitions $2 \rightarrow 1'$, $2'$, and $3'$ of the $^{85}$Rb D2 lines instead of $F_g=3$. Figure 3 presents a comparison of the calculated and measured spectra considering (a) pure two-level resonant transitions, i.e., $2 \rightarrow 1'$, $2 \rightarrow 2'$ and $2 \rightarrow 3'$; (b) a transition resonant at $2 \rightarrow 1'$ with neighboring hyperfine transitions of $2 \rightarrow 2'$ and $3'$, a transition resonant at $2 \rightarrow 2'$ with neighboring hyperfine transitions of $2 \rightarrow 1'$ and $3'$, a transition resonant at $2 \rightarrow 3'$ with neighboring hyperfine transitions of $2 \rightarrow 1'$ and $2'$; (c) experimentally measured spectra for the resonant transitions of $2 \rightarrow 1'$, $2 \rightarrow 2'$ and $2 \rightarrow 3'$ for both weak (50 $\upmu$W) and strong (4 mW) coupling powers as shown in the top and bottom traces, respectively.

The calculation for a DTLS in the low-power regime clearly reproduces ultra-narrow EIT resonances, as expected, at the $2 \rightarrow 1'$, $2 \rightarrow 2'$ and $2 \rightarrow 3'$ transitions, as shown in the upper traces in Fig. 3. The obtained EIT spectra differ in relative amplitude with the dominant EIT amplitude at the $2 \rightarrow 1'$ transition, as shown in the upper traces in Fig. 3a. The obtained linewidth resolutions of the EIT spectra are 90 kHz, 60 kHz, and 130 kHz at the $2 \rightarrow 3'$, $2'$, and $1'$ hyperfine transitions, respectively.

The EIT spectra calculated considering all the neighboring states near the resonant state are similar in amplitude and contrast owing to the strong EIT amplitude at the $2 \rightarrow 1'$ neighboring hyperfine transition, as shown in the upper traces of Fig. 3b. The trend of the measured spectra, shown in Fig. 3c, exhibits a striking similarity in terms of amplitude and a contrast that matches well with the calculation, as shown in Fig. 3b, when considering neighboring states. The resolved linewidth in the low-coupling-power regime is approximately 114 kHz across the $2 \rightarrow 3',2'$, and $1'$ transitions for all the neighboring calculations, as shown in Fig. 3b; the experimentally determined resolved linewidths (Fig. 3c) are 157 kHz, 158 kHz, and 163 kHz at the $2 \rightarrow 3',2'$, and $1'$ transitions, respectively.

In the strong-coupling-power regime (the lower traces of Fig. 3), the calculation considering the resonant states shows an increase in the amplitudes and linewidths of the obtained spectra at the $2 \rightarrow 1'$ and $2 \rightarrow 2'$ hyperfine transitions. However, in violation of condition (3), a weak-amplitude EIA with a linewidth resolution of 285 kHz is obtained at the $2 \rightarrow 3'$ hyperfine transition, as highlighted in the inset image of the lower traces in Fig. 3a.

Asymmetric EIT spectra of relatively similar amplitudes and linewidth resolutions are obtained owing to the contributions of all the neighboring hyperfine states with the resonant transition, as shown in the lower traces in Fig. 3b; this agrees with the trend of the measured spectra, shown in the lower traces in Fig. 3c. However, the experimentally measured spectra at higher powers do not reflect the fine contrast, unlike the calculation with all the neighboring transitions shown in the lower traces in Fig. 3b. There is a dent on the right side of the calculated spectrum at higher power regime, but we were not able to observe this dent in the experiment. In a preliminary study we find that the transit-relaxation rate affects the absorption profiles substantially. We will perform a detailed experimental and theoretical study on the effect of the transit-relaxation rate on the transmission spectra. The resolved linewidths are 388 kHz, 407 kHz, and 435 kHz (441 kHz, 418 kHz, and 479 kHz) when using all the neighboring calculations (experiment).

The sign-inverting anomalies present at open transitions $3 \rightarrow 3'$ and $3 \rightarrow 2'$ in the previous cases, as shown in Fig. 2b,c, are not relevant to the $2 \rightarrow 1',2'$, and $3'$ group of spectrally resolved transitions, because EIT resonances at the $2 \rightarrow 3',2'$, and $1'$ transitions are expected. Instead, anomalies in the relative strength of the amplitudes and linewidths at $2 \rightarrow 3',2'$, and $1'$ are evident in the calculation considering all the neighboring transitions and in the experiment, as shown in the lower traces in Fig. 3b,c, respectively.

From both cases of $F_g=2$ and 3, we observe that the power ratios between the coupling and probe beams, the openness of the excited state, and effects of neighboring states due to Doppler broadening are crucial factors in enhancing or reducing the coherence effects associated with transformations between EIA and EIT.

## Discussion

In this study, we solved the optical Bloch equations with and without neighboring hyperfine states for all hyperfine transitions of the $^{85}$Rb D2 lines both in weak- and strong-coupling-power regimes while considering the Doppler effect in an ordinary vapor cell. The calculated spectra matched well with the observed spectra. Theoretical models based on time-dependent density matrix equations of a degenerate (a) two-level (resonant transitions) and (b) multilevel system including all neighboring hyperfine transitions were considered for calculating coherence effects, such as EIT and EIA, using thermal averaging over the Doppler profile. EITs without neighboring effects in the case of open $3 \rightarrow 2'$ and $3'$ transitions, i.e., a DTLS that violates Lezama’s EIA conditions, transform into genuine and symmetric EIAs owing to a strong $3 \rightarrow 4'$ EIA line with Doppler broadening in the weak coupling and probe power regime. This phenomenon had remained theoretically unexplained and had not been observed previously in the case of $^{85}$Rb D2 lines. Moreover, asymmetric and split EIAs were observed from calculated weak EITs in a DTLS in a weak probe and strong-coupling-power regime. The previously reported EIA resonances without clear quantitative explanations for open systems of the D1 lines of $^{85}$Rb and $^{87}$Rb, which violate Lezama’s EIA conditions (2) and (3), are merely enhanced absorption phenomena governed by a strong coupling power^7,8,11,13,16–18^. These are different from the EIAs in the same transition configuration of $F_e=F_g-1$ and $F_g$ observed and explained in this study using weak coupling-probe powers.

The EITs without neighboring effects in the DTLS in the case of $2 \rightarrow 1'$ ($2 \rightarrow 2'$) are not transformed, owing to the $2 \rightarrow 2'$ ($2 \rightarrow 1'$) EIT line with the Doppler broadening in the weak probe and strong-coupling-power regime. In contrast, weak EIAs at the $2 \rightarrow 3'$ transition in the weak probe and strong-coupling-power regime, which violate Lezama’s EIA conditions because of openness, transform into EITs. Lezama’s EIA conditions in the cycling case of $3 \rightarrow 4'$ with $F_g < F_e$ are also confirmed in strong coupling and weak probe regimes. With the comparisons of the calculated absorption profiles between a DTLS without neighboring states and all levels with neighboring states, the power ratio between the probe and coupling beams, the openness of the excited state, and effects of neighboring states due to Doppler broadening are crucial factors that enhance or reduce the coherence effects associated with transformations between EIA and EIT.

## Methods

**Figure 4:**
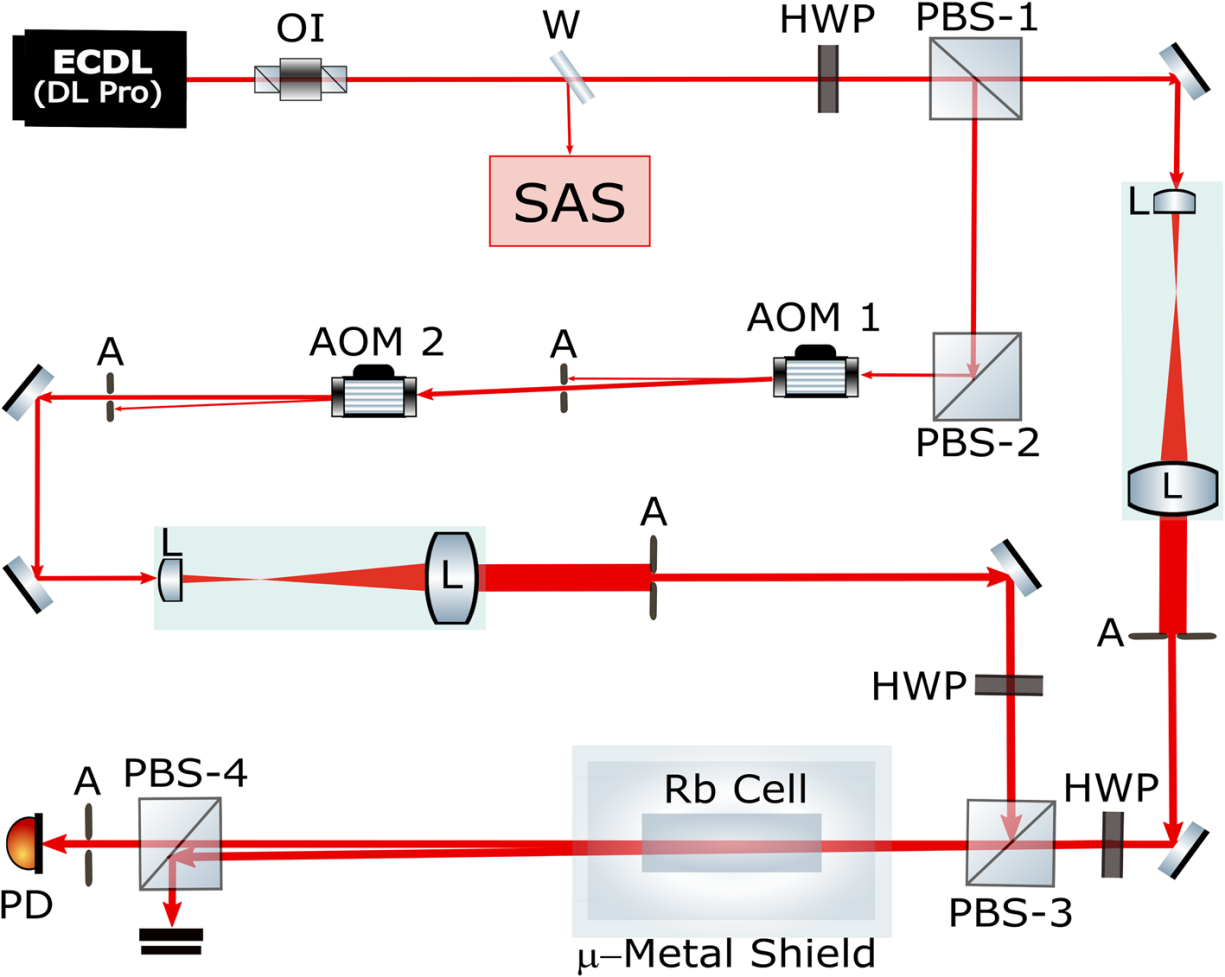
Experimental setup. Component symbols: OI: optical isolator; W: window; SAS: saturation absorption spectroscopy; HWP: half-wave plate; PBS: polarizing beam splitter; A: aperture; QWP: quarter-wave plate; L: lens; PD: photodiode; AOM: acousto-optic modulator. Figure 4 is drawn by the first author (Zeeshan Ali Safdar Jadoon) using free open-source scalable vector graphics editor Inkscape (Version: 1.0.2-2) available at: https://inkscape.org/.

The coupling and probe beams were generated using a single laser (DLPro, Toptica Inc.) combined with two AOMs in a single-pass configuration, as shown in Fig. 4. A combination of a half-wave plate (HWP) and polarizing beam splitter 1 (PBS-1) controls the ratio between the intensities and polarizations of the coupling and probe beam. The probe and coupling beams were linearly polarized in orthogonal directions. A weak-probe-beam power of 15 $\upmu$W and a coupling beam with a scanning frequency of 5 MHz at a low coupling power of 50 $\upmu$W and strong coupling power of 4 mW were used. The coupling and probe beams were expanded by five times to obtain a uniform intensity across a 4-mm diameter. The coupling beam with the scanning detuning ($\Delta$) from AOM-2 was combined with the weak probe at PBS-3 and co-propagated with the orthogonal linear polarizations while maintaining an angle of intersection of approximately 0.1 mrad through the vapor cell, which was shielded with five layers of $\mu$-metal sheets. PBS-4 eliminated the coupling beam before the detection of the probe intensity at a photodiode (PD). The probe laser frequency was locked to a desired $5S_{1/2} \rightarrow 5P_{3/2}$ transition of the $^{85}$Rb D2 hyperfine line, i.e., $3 \rightarrow 2'$, $3 \rightarrow 3'$, $3 \rightarrow 4'$, $2 \rightarrow 1'$, $2 \rightarrow 2'$, or $2 \rightarrow 3'$ using a saturation absorption spectroscopy (SAS) setup. The coupling laser frequency was scanned around each resonance line. The experiments were performed with $^{85}$Rb atoms in a vapor cell at a temperature of 20 $^\circ$C.
